# RNA sequencing of chorionic villi from recurrent pregnancy loss patients reveals impaired function of basic nuclear and cellular machinery

**DOI:** 10.1038/srep38439

**Published:** 2016-12-08

**Authors:** Siim Sõber, Kristiina Rull, Mario Reiman, Piret Ilisson, Pirkko Mattila, Maris Laan

**Affiliations:** 1 Human Molecular Genetics Research Group, Institute of Molecular and Cell Biology, University of Tartu, Riia St. 23, 51010 Tartu, Estonia; 2 Department of Obstetrics and Gynaecology, University of Tartu, L. Puusepa St. 8, Tartu 51014, Estonia; 3 Women’s Clinic of Tartu University Hospital, L. Puusepa St. 8, Tartu 51014, Estonia; 4 Department of Genetics, United Laboratories of Tartu University Hospital, L. Puusepa St. 2, Tartu 51014, Estonia; 5 The Institute for Molecular Medicine Finland (FIMM), Tukholmankatu 8, Helsinki FI-00014 Finland; 6 Finnish Red Cross Blood Service (FRCBS), Kivihaantie 7, Helsinki FI-00310, Finland; 7 Institute of Biomedicine and Translational Medicine, University of Tartu, Ravila St. 19, 50412 Tartu, Estonia

## Abstract

Recurrent pregnancy loss (RPL) concerns ~3% of couples aiming at childbirth. In the current study, transcriptomes and miRNomes of 1^st^ trimester placental chorionic villi were analysed for 2 RPL cases (≥6 miscarriages) and normal, but electively terminated pregnancies (ETP; n = 8). Sequencing was performed on Illumina HiSeq 2000 platform. Differential expression analyses detected 51 (27%) transcripts with increased and 138 (73%) with decreased expression in RPL compared to ETP (DESeq: FDR *P* < 0.1 and DESeq2: <0.05). RPL samples had substantially decreased transcript levels of histones, regulatory RNAs and genes involved in telomere, spliceosome, ribosomal, mitochondrial and intra-cellular signalling functions. Downregulated expression of *HIST1H1B* and *HIST1H4A* (Wilcoxon test, fc≤0.372, *P*≤9.37 × 10^−4^) was validated in an extended sample by quantitative PCR (RPL, n = 14; ETP, n = 24). Several upregulated genes are linked to placental function and pregnancy complications: *ATF4, C3, PHLDA2, GPX4, ICAM1, SLC16A2*. Analysis of the miRNA-Seq dataset identified no large disturbances in RPL samples. Notably, nearly 2/3 of differentially expressed genes have binding sites for E2F transcription factors, coordinating mammalian endocycle and placental development. For a conceptus destined to miscarriage, the E2F TF-family represents a potential key coordinator in reprogramming the placental genome towards gradually stopping the maintenance of basic nuclear and cellular functions.

Pregnancy loss, defined as an expulsion of the conceptus before 22 weeks of gestation, is the most common pregnancy complication. One in ten clinically confirmed pregnancies results in miscarriage. In fact, already prior to implantation stage ~30% of human conceptions are lost and another ~30% are detected only as ‘biochemical pregnancies’ (positive circulating hCG test), which fail to develop further^1^. Recurrent pregnancy loss (RPL)^2^, concerning ~3% of couples aiming at childbirth, is generally defined as ≥2 or ≥3 consecutive pregnancy losses of a couple^3^,^4^,^5^. Whereas the majority of sporadic cases of pregnancy loss (~75%) are due to chromosomal aneuploidies arising from occasional non-inherited nondisjunction events, the known causes behind RPL include parental chromosomal aberrations, maternal thrombophilic and (auto) immune disorders, endocrine disturbances, uterine abnormalities and infections^1^,^2^,^6^,^7^. Still, couples with idiopathic RPL without any clear cause represent 25–50% of all cases. A longitudinal study from the Danish Recurrent Pregnancy Loss Clinic showed that even after being referred to a specialized RPL clinic, 33% of the RPL couples remain childless after 5 years^8^. Epidemiological studies have revealed that a subsequent pregnancy after a series of miscarriages has an increased risk to preterm birth, stillbirth, preeclampsia and fetal growth restriction^9^,^10^. Taken together – as RPL represents an unrecognized common disorder with immediate and long-term consequences on an entire family, there is an urgent need to uncover novel mechanisms behind its occurrence with the ultimate goal to utilize this knowledge in clinical management.

Capturing gene expression profiles and biological pathways involved in failed pregnancy may assist in pinpointing novel biomarkers or therapeutic targets potentially applicable in clinical conditions for the benefit of RPL patients. There are limited studies investigating transcriptome-wide gene expression signatures linked to the disturbances at the maternal-fetal interface, possibly contributing to abnormal placental development and/or reflecting the biological mechanisms behind RPL. The two seminal studies using gene expression microarray profiling of placental samples from RPL cases detected no differentially expressed genes with highly significant statistical support. The first study detected 133 genes showing a trend (fold change (fc) >2; nominal *P*-value < 0.05) for decreased and 22 genes for increased expression in the deciduas (maternal side of the placenta) of RPL patients compared to uncomplicated pregnancy^11^. The study by our team identified only 27 genes (fc, 1.4 to 4.1; *P*-value < 0.05) with a trend for altered expression in the RPL placental tissue^12^. Both reports highlighted an enrichment of dysregulated genes involved in cell communication and signalling, inflammatory and immune response.

Compared to microarrays, RNA sequencing (RNA-Seq) provides substantially higher resolution to capture the entire transcriptome, capable for precise measurement of the level of all protein-coding and non-coding (ncRNA) transcripts. RNA-Seq has several other advantages, such as higher statistical power due to >10–15 times wider dynamic range to quantify gene expression level compared to microarrays^13^. We have previously applied RNA-Seq to investigate placental differential gene expression signatures in prevalent adverse pregnancy outcomes at term^14^. The analysis convincingly showed that the placental transcriptome in late gestational complication preeclampsia is clearly distinct not only from normal pregnancy, but also from other common adverse pregnancy outcomes at term. The current study aimed to bring novel insights behind the molecular mechanisms and involved pathways leading to pregnancy loss. We utilized RNA-Seq and miRNA-Seq to profile the transcriptome of the first trimester placental chorionic villi in cases of RPL compared to normal, but electively terminated (ETP) pregnancies. Our data shows that in the placentas from RPL cases, the majority of differentially expressed genes are downregulated. These genes are explicitly involved in the basic machinery required for replication and chromatin integrity, transcription and RNA processing, maintenance of mitochondria and other essential genome functions required in the process of rapid cellular proliferation and differentiation critical in early placental development. The identified genes with increased transcript levels confirmed several loci previously linked to placental malfunction in RPL pregnancies. Most importantly, a large fraction of differentially expressed genes in RPL possess binding sites for E2F transcription factors known to be involved in regulating the replication machinery of the mammalian endocycle – a key process to guarantee normal trophoblast proliferation and invasion, placental development, and fetal viability.

## Results

### RNA-Seq dataset of first trimester chorionic villi from normal and RPL pregnancies

We performed whole transcriptome (RNA-Seq) and miRNome (miRNA-Seq) sequencing for chorionic villi dissected from the placental samples representing first trimester human pregnancy. The study sample included two cases of recurrent pregnancy loss (RPL1, female fetus, fetal death at the 44^th^ gestational day; RPL2, male, 67^th^ g. d.) and a control group comprised of clinically normal, electively terminated pregnancies (ETP; n = 8; 3 male, 5 female fetuses; 51 to 81 g. d.) (Table 1). Before the event, the two RPL cases had experienced five and six clinically confirmed pregnancy losses, respectively. At the index pregnancy, the placental sampling was conducted along with the surgical removal of conceptus shortly after detection of fetal death (see **Methods**; **Supplementary Methods**).

Total RNA extracted from chorionic villous samples and depleted of ribosomal RNA was subjected to previously reported^14^ RNA-Seq pipeline providing 46-base pair (bp) paired-end reads (Illumina HiSeq 2000). After filtering the raw reads, the transcriptomes of analysed chorionic villous samples in the RPL samples yielded 41.3 and 63.3 million paired-end reads (Supplementary Fig. S1a). Individual transcriptomes of the 8 ETP samples yielded on average 42.0 million paired-end reads (range: 28.1–48.2 million). The reads were aligned to the human genome with an average success rate of 85.2% (range: 80.2–91.5%; Supplementary Fig. S1b). The complete RNA-Seq dataset over the sequenced 1^st^ trimester chorionic villous samples (n = 10) was comprised of 31.8 billion aligned bases and in total providing ~199 fold mean coverage over exonic regions (exonic coverage per sample: median 19.3, range 11.3–36.0 fold; Supplementary Fig. S1c,d). RNA-Seq datasets of all samples exhibited similar distribution and quantity of read counts (Supplementary Fig. S1f). The remaining clinical material enabled to perform miRNA sequencing for five of the eight ETP samples and both RPL samples (Supplementary Fig. S1e). Per sample, on average 21.7 million reads (range: 5.7–46.2 million) were produced with high mapping success to the human genome (mean: 95.6%; range: 88.8-98.8%). Of the mapped reads, mean 14.7% (range: 9.8-20.5%) were aligned to 732 mature microRNAs, generating the miRNA-Seq dataset investigated in the current study.

### Underrepresentation of ncRNAs and histones in the placental transcriptome of RPL cases

**Figure 1:**
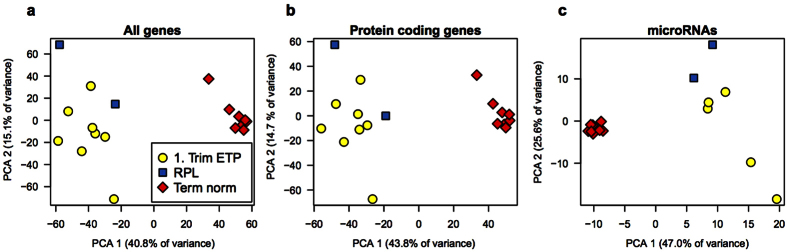
Principal component analysis (PCA) of normalized read counts after variance stabilizing transformation in DESeq2. The first two principal components are plotted for **(a)** All genes (n = 14,767), **(b)** protein coding genes (n = 12,643) and **(c)** microRNAs (n = 335). Transcripts with mean read count < 50 were excluded.

The first principal component (PC1, 40.8% of variance) of the PCA across the placental transcriptomes of ETP (n = 8), RPL (n = 2) and normal gestations at term (Term norm, n = 8; dataset from^14^) separated clearly the 1^st^ and 3^rd^ trimester placental tissues (n = 14,767 analyzed transcripts; Fig. 1a). The same applies for the expression profile of placental protein encoding genes (n = 12,643; Fig. 1b) and miRNAs (n = 355; Fig. 1c), which appeared to be distinct for the early and late pregnancy (PC1 43.8% and 47.0% respectively). On the PCA plot, the transcriptomes of the 1^st^ trimester chorionic villi from the RPL and the ETP cases did not form clearly identifiable clusters. However, the microRNA profiles of RPL and ETP samples appeared to be separated by the second principal component, representing 25.6% of overall variance (Fig. 1c).

**Figure 2:**
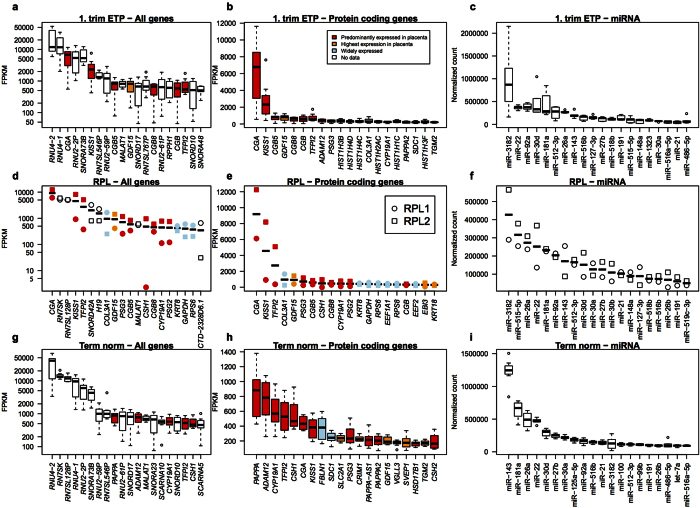
Expression levels of top 20 most abundant transcripts (**a,d,g**) logarithmic scale), protein-coding genes (**b,e,h**) linear scale) and microRNAs (**c,f,i)**, linear scale; expressed in normalized read counts) in samples representing 1^st^ trimester chorionic villi from normal (ETP, n = 8; (**a,b**) and recurrent pregnancy loss (RPL, n = 2; (**d,e,f**) placentas compared to normal term placentas (n = 8; (**g,h,i**). Annotation of placental transcripts detected and quantified by the RNA-Seq pipeline was based on ENSEMBL v67 database. Gene expression levels are expressed in FPKM (Fragments per kilobase of exon per million fragments mapped) as determined by cufflinks v 2.0.2; miRNA expression levels are given as DESeq2 normalized read counts. Data on the enrichment of gene expression in the placenta compared to other tissues was derived from Protein Atlas v12. Expression profile across tissues for noncoding RNAs was not available.

In the ETP placental samples, 13 of the 20 most highly expressed transcripts represented various small non-coding RNAs (ncRNAs), essential for the key nuclear and cellular functions such as pre-RNA and ribosomal RNA splicing (*RNU4, RNU2* snRNA genes) and processing (*SNORA* and *SNORD* snoRNAs families), signal recognition particle RNAs involved in translation (7SL ncRNA family; Fig. 2a). Another class of prevalent transcripts in the 1^st^ trimester placental sample is responsible for the adequate secretion of placental hormones, e.g. *CGA* and *CGB* genes encoding for the alpha and beta-subunit of hCG, and *KISS1* gene encoding kisspeptin. This transcriptome profile is consistent with our recently published data on term placentas^14^, where also the majority of the highly expressed genes encoded either regulatory ncRNAs or placental hormones (Fig. 2g). The profile of genes with the top expression level in the RPL placentas differed significantly from normal pregnancies for the proportion of ncRNAs (Fig. 2d; Supplementary Data S1). In the RPL samples only 23 of the 200 most highly expressed transcripts represent various ncRNAs compared to 60/200 for the ETP samples (Fisher’s exact test, *P*-value = 2.46 × 10^−6^). The top-200 most abundantly expressed genes in the RPL placentas miss completely the small nuclear genes *RNU1–4*, which are essential components of the spliceosome complex.

The placental transcriptome of the RPL and ETP cases differed also for highly expressed protein coding genes (Fig. 2b,e). In addition to the abundance of transcripts encoding placenta-specific hormones (*CGA, CGB, KISS1, ADAM12, PSG3, CYP19A1*), the chorionic villous tissue in the 1^st^ trimester normal pregnancy was also characterized by high expression of histone encoding genes. Histones represented 36 of 200 genes with the highest expression level in the ETP samples compared to only one gene in the RPL samples (Fisher’s exact test, *P*-value = 0). As in term placentas the processes of cellular proliferation, replication and mitosis are slowed down, the proportional expression levels of histones and other basic components of the chromatin formation and nuclear machinery have dropped (Fig. 2h).

In contrast to the distinct set of the top-expressed genes in the RNA-Seq analysis, the majority of most abundantly expressed microRNAs overlap between the chorionic villous samples from the ETP and RPL cases (17/20 in the top list; Fig. 2c,f; Supplementary Data S2a). Interestingly, the top-20 lists in both ETP and RPL samples are enriched for microRNA, which belong to two placenta-specific imprinted miRNA clusters: paternally expressed C19MC (miR-512–3p, miR-515-5p, miR-516a-5p, miR-516b, miR-518b, miR-519c-3p) and maternally expressed C14MC (miR-127-3p)^15^. Among the top-100 microRNAs expressed in 1^st^ trimester chorionic villi, more than one third (ETP: 38; RPL: 37 miRNAs) map to these placental microRNA clusters, evolved in primates (C19MC) or in mammals (C14MC, miR-371-3 cluster) (Supplementary Data S2b,c). The list of miRNAs with the highest placental expression overlaps between 1^st^ trimester and term pregnancy placentas, although gestational dynamics of their transcript levels were observed (Fig. 2I; Supplementary Data S2d). In early pregnancy chorionic villi, miR-3182 was the highest expressed microRNA in both, the ETP and RPL groups. At term, the predominantly expressed microRNA was miR-143 exhibiting about 2-fold higher transcript level compared the next three top-expressed miRNAs (miR-181a, miR-26a, miR-22). The detected transcript levels of all other analyzed miRNAs were substantially lower. The placenta-specific miR-371-3 cluster was expressed highly in ETP and RPL, but its estimated expression level was reduced more than 4-fold at term.

### Transcriptome profile in the RPL placentas reflects malfunction of basic nuclear processes and the organismal feedback mechanisms to rescue the fetus

**Figure 3:**
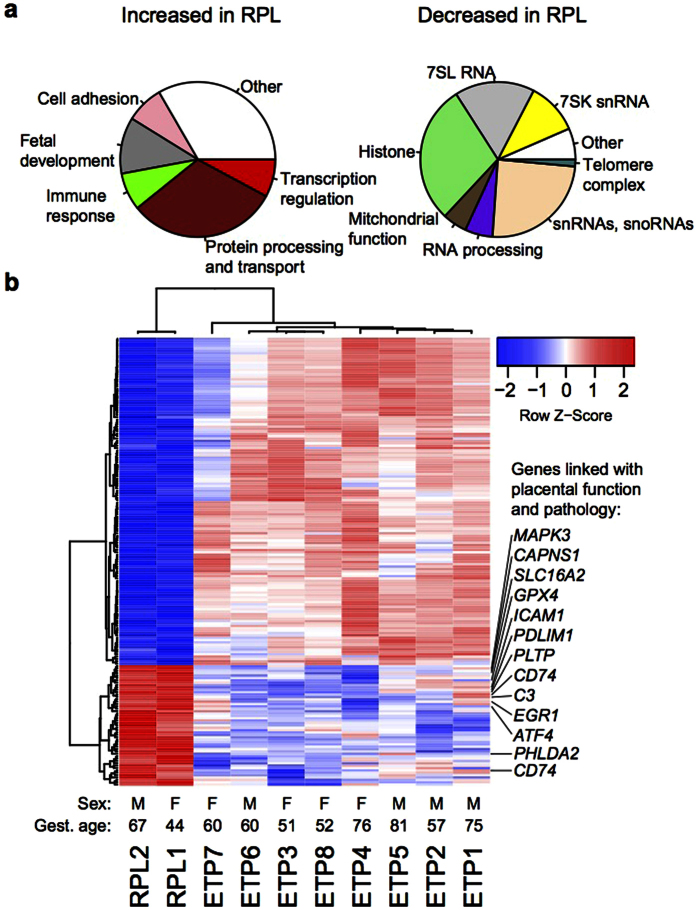
Genes with significantly (FDR adjusted *P* < 0.1 for DESeq and P < 0.05 for DESeq2; n = 189) altered expression levels in recurrent pregnancy loss (RPL, n = 2) compared to electively terminated 1st trimester pregnancies (ETP, n = 8). (**a)** Pie charts of functional categories of genes with elevated (n = 51, left) and reduced (n = 138, right) expression. (**b)** Heatmap with hierarchical clustering based on transformed read counts of the differentially expressed genes. Gene expression levels were subjected to variance stabilizing transformation in DESeq2 and standardized by subtracting the mean expression across all samples from its value for a given sample and then dividing by the standard deviation across all the samples. This scaled expression value, denoted as the row Z-score, is plotted in red-blue color scale with red indicating increased expression and blue indicating decreased expression. Hierarchical clustering of genes (rows) and samples (columns) was based on Pearson’s correlation. Hierarchical clustering trees are shown for the analyzed samples (top) and genes (left). For each sample newborn sex (M, male; F, female), and gestational age in days at sampling are given below the heatmap. Genes with prior evidence in the scientific literature for the involvement in placental function and pathology are highlighted on the right.

Transcriptome of chorionic villi from the 1^st^ trimester RPL (n = 2) compared to ETP (n = 8) cases was characterized by a major shift in the expression levels of high number of genes (Fig. 3; Supplementary Data S3a). Differential expression of 189 genes matched the stringent significance criteria applied in this study (false discovery rate (FDR) adjusted *P* < 0.1 for DESeq and *P* < 0.05 for DESeq2; details in ‘**Methods’; Supplementary Methods**). Of these genes, 27% (n = 51) showed significantly higher and 73% (n = 138) lower transcript levels in RPL compared to ETP placentas. Notably, the functional categories of over- and under-expressed genes in RPL were distinct. Significantly upregulated loci are mostly protein encoding genes, which are involved in the protein processing/transport (n = 15; 29% of upregulated transcripts), fetal development (n = 6; 12%), cell adhesion, immune response and transcriptional regulation (n = 4; each 8%; Fig. 3a). Among them the genes *ATF4*^16^,^17^, *C3*^18^,^19^*, PHLDA2*^20^, *GPX4*^21^ and *ICAM1*^22^ have been previously linked with the pathogenesis of pregnancy loss. Additionally, *GPX4* has been implicated in preeclampsia^23^, *PHLDA2*^24^,^25^,^26^ and *SLC16A2*^27^,^28^ in fetal growth restriction, *CD74* and *LAPTM5* in preterm premature rupture of membranes^29^ (details and full list of references in Supplementary Data S3b). Several short-listed genes function in trophoblast invasion, proliferation and syncytialization processes (*EGR1, PDLIM1, MAPK3*). In total, 13 of 51 genes exhibiting increased expression in the RPL samples were supported by previous evidence for the involvement in placental function and/or pathology (Fig. 3a).

In contrast, the majority of genes exhibiting decreased expression represent various snRNAs/snoRNAs (n = 25; 18% of down-regulated transcripts), as well as ncRNAs involved in transcriptional, telomere, spliceosome and cellular protein traffic complexes (in total, n = 48; 35%) and components responsible for the basic cellular apparatus for chromatin assembly (histone proteins, n = 29; 21%) and mitochondrial function (n = 7; 5%). These are critical processes to guarantee rapid proliferation and invasion of trophoblast in early pregnancy.

Differential expression analysis between the miRNA-Seq datasets of RPL (n = 2) and ETP (n = 5) samples identified no large disturbances in pregnancy loss samples (Supplementary Data S4). Among the microRNAs entering differential expression testing (n = 312), only miR-3168 showed statistically significant, ~ 8-fold elevated transcript levels in RPL compared to ETP (DESeq: FDR adjusted *P* = 0.018; DESeq2: FDR *P* = 8.81 × 10^−4^). DESeq2 algorithm detected decreased and increased expression in RPL samples for additional 25 and 27 miRNAs, respectively. Several of these miRNAs have been shown to modulate the expression of tumor suppressor genes involved in proliferation and invasion (e.g. miR-1260b, miR-193a-3p, miR-494, miR-142-3p).

### Concordant contribution of both RPL samples to differential expression outcome

As the sample size of our differential expression testing was small (RPL n = 2; ETP, n = 8), we assessed the robustness of its outcome by analysing the potential individual contribution of the two RPL samples. First, when we assessed the correlation of normalized gene expression read counts between the two RPL samples, the generated RNA-Seq (r^2^ = 0.92) and miRNA-Seq (r^2^ = 0.94) datasets showed high concordance (Supplementary Fig. S2). Next, we performed differential expression tests comparing the transcriptome of both RPL RNA-Seq datasets separately with the ETP group (Supplementary Data S5a,b). Gene expression levels for a very early miscarriage case RPL1 (44 g.d.; female) differed significantly from the ETP samples (median gestational age 60 days) for as many as 483 transcripts. The transcriptome of chorionic villi from the RPL2 case representing later gestational age (67 g.d., male) showed differential expression for 196 transcripts. Both separate lists of differentially expressed transcripts included histone encoding genes (n = 48 and n = 46, respectively) and various ncRNAs (n =  69; n = 66) in the same abundance as the RPL *vs* ETP group comparisons. In the mutual comparison of the RPL1 and RPL2 transcriptomes, 11 of the top 100 transcripts with largest difference in expression levels were sex-chromosomal genes referring to the different sex of the samples (Supplementary Data S5c). The remaining list of 89 transcripts included no histone genes, no ncRNA (except for a single lincRNA), no genes involved in mitochondrial function. Most probably, differences in measured gene expression levels between RPL1 and RPL2 reflect gestational dynamics in transcription regulation and stochastic differences between samples (statistical significance of these differences cannot be reliably estimated). In progression of pregnancy from 44 to 67 gestational days, 61/100 differentially expressed genes showed increased expression and several of them encoded critical placental hormones (*CSH1, GH2, PAPPA2, HSD11B2, LGALS14*).

### Confirmation of RNA-Seq results by Taqman RT-qPCR in an extended dataset

**Figure 4:**
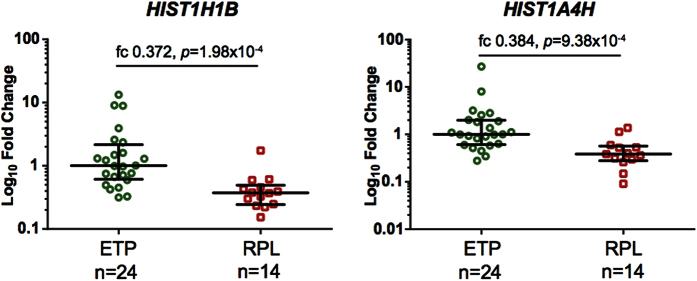
Significantly decreased expression of *HIST1H1B* and *HIST1H4A* in placental tissue from the cases of recurrent pregnancy loss (RPL, n = 14) compared to uncomplicated, but electively terminated pregnancies (ETP, n = 24). Placental gene expression levels were measured using Taqman RT-qPCR (*HIST1H1B* assay ID: Hs00271207_s1; *HIST1H4A* assay ID: Hs00747492_s1). The median expression level of the ETP group was selected as a calibrator and the respective relative mRNA expression levels are shown on logarithmic scale. Dots represent data of each patient; the bars denote median and the 25^th^ and 75^th^ percentiles. Fold change (fc) was calculated as the difference of mean relative expression value of RPL versus ETP group; *P*-values were estimated by Wilcoxon test.

Validation of differential gene expression for selected genes was performed using Taqman RT-qPCR analysis in an extended placental sample-set (n = 14 RPL cases, 24 ETP controls; Supplementary Table S1 and S2). Statistically significant reduction in expression level in cases of RPL compared to ETP controls was robustly validated for the two analyzed histone-encoding genes, *HIST1H1B* and *HIST1H4A* (Wilcoxon test, fold change (fc) = 0.372, *P* = 1.97 × 10^−4^ and fc = 0.384, *P* = 9.37 × 10^−4^, respectively; Fig. 4). When the analysis was adjusted for gestational age, the statistical significance of differential expression between ETP and RPL samples level remains statistically significant (logistic regression, *HIST1H1B: P* = 4.8 × 10^−3^; *HIST1H4A: P* = 6.3 × 10^−3^).

Additionally, Taqman RT-qPCR analysis showed a trend for an increased expression of the *Complement Component 3 (C3*) gene, concordant with the RNA-Seq dataset (fc = 3.57, *P* = 0.162; Supplementary Fig. S3). However, this outcome did not reach statistical significance possibly due to differences in the study material composition (RNA-Seq: microdissected chorionic villi; Taqman RT-qPCR: placental samples comprised of variable cell types). The validation experiments did not confirm increased expression of *CAPNS1* and *PLTP* in extended RPL placental tissues.

### Limited effect of maternal age on placental gene expression in the first trimester ETP samples

**Figure 5:**
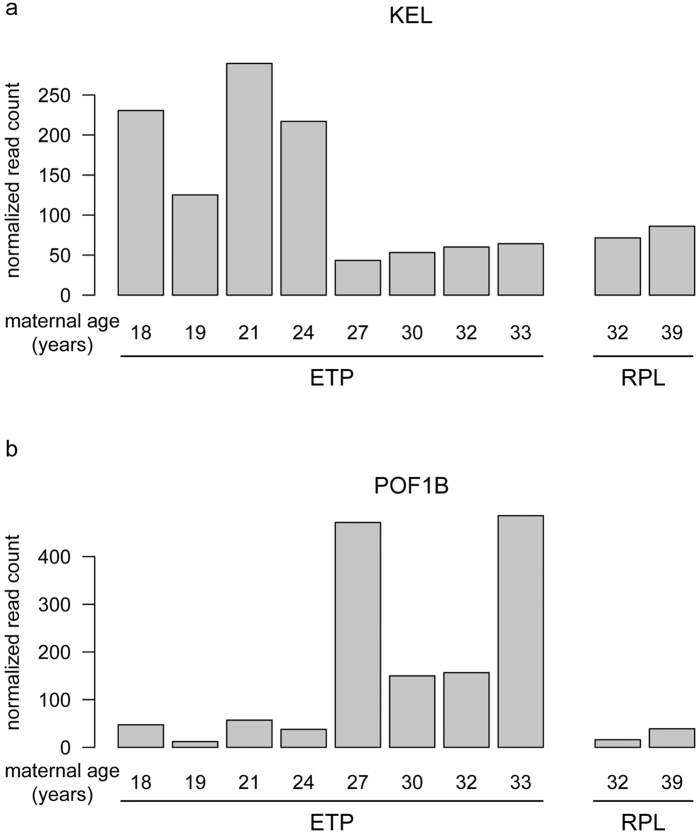
Maternal age dependent differential expression in the placenta representing uncomplicated early pregnancy (ETP group, n = 8) was detected for only two genes (*KEL, POF1B*; DESeq2: FDR P < 0.05; Supplementary Data S6). **(a)** *KEL (Kell blood group, metallo-endopeptidase*) and **(b)** *POF1B (Premature ovarian failure protein 1B*) gene expression levels are presented as normalized read counts for individual chorionic villous samples in the ETP (left) and RPL (right) groups. Maternal ages in years are given for each analysed sample. ETP, electively terminated pregnancy; RPL, recurrent pregnancy loss.

As the average maternal age of the RPL samples (mean: 35.5; median: 35.5; range: 32–39 years) was higher compared to the ETP controls (mean: 25.5; median 25.5; range: 18–33), we investigated a potential confounding effect of the maternal age on placental gene expression in early pregnancy. Differential expression was tested between the ETP subgroups representing young (18, 19, 21 and 24 years) and early middle-aged women (27, 30, 32 and 33 years). No genes corresponded to the robust differential expression criteria applied in the current study (FDR *P* < 0.1 for DESeq and *P* < 0.05 for DESeq2). In the analysis using DESeq programme, none of the tests reached FDR levels below one (Supplementary Data S6). Differential expression testing implemented in DESeq2 highlighted two genes: *KEL* (FDR *P* = 5.40 × 10^−4^) and *POF1B* (FDR *P* = 1.24 × 10^−3^) (Fig. 5; Supplementary Data S6). Both genes exhibit low to moderate placental expression (mean read counts < 200) and no previous studies have assessed their expression in the human placenta. The *KEL* gene encodes the KELL blood group antigen (OMIM: #110900) and exhibited reduced expression in older mothers and RPL patients (Fig. 5a). *POF1B (Premature ova-rian failure protein 1)* is mostly expressed in epithelia and specifically in skin epidermis^30^ and low transcript levels have been also detected in female reproductive tissues, cervix, fallopian tube, placenta^31^,^32^. Mutations in *POF1B* have been associated with premature ovarian failure^33^. In chorionic villous samples, POF1B exhibited elevated expression in older ETP mothers, but not in mothers with RPL (Fig. 5b). Based on these findings, supported by our previous RNA-Seq study in 40 term placentas^14^, we conclude that a confounding effect of maternal age on placental gene expression, if present, is limited.

### Potential key role of E2F transcription factor family in triggering the fetal programming towards pregnancy loss

To further functionally characterize the biological pathways involved in altered transcriptome profile in the RPL compared to the ETP placentas, we performed gene set enrichment analysis for the list of differentially expressed genes. As expected, among the down-regulated genes an extreme enrichment for functional categories including histones was observed, such as ‘Nucleosome’ (GO:0000786; *P*  = 2.34 × 10^−53^; 32.1% of the genes in the category), ‘Nucleosome assembly’ (GO:0006334; *P* = 3.27 × 10^−34^; 18.8%) and histone H4-K20 demethylation (GO:0035574; *P*  = 6.46 × 10^−10^; 43.8%) (Table 2; Supplementary Data S7). Genes involved in a large pathway ‘Extracellular region’ (GO:0005576; *P*  = 7.36 × 10^−14^) represent 2/3 of the full list of independent annotated genes entering the functional profiling analysis. Whereas the list of transcripts in this category with decreased expression is comprised of histones and loci involved in mitochondrial function, the list of transcripts with increased expression level are encoded by several genes previously implicated in placental function, pregnancy loss and/or complications, such as *CD74, ICAM1, PLTP, MAPK3, C3, GPX4*.

Importantly, a few transcription factors (TF) emerged as potential key players in programming the trophoblastic genome and cells towards discontinuation of the pregnancy. Notably, 11 of 15 TF-binding sequence motifs, which were enriched in the promoter regions of differentially expressed genes in RPL chorionic villous samples, represented binding sites for various E2F TF family members or E2F:DP-1 heterodimer complexes (Table 2; Supplementary Data S7). The most significant enrichment was detected for the E2F motif ‘NCSCGCSAAAN’ (TF:M00919_1; *P* = 5.31 × 10^−6^; 3% of pathway genes; 14.7% of query genes). The largest fraction of genes with either increased or decreased transcript levels carried the binding sites for E2F-1 (motif ‘NKTSSCGC’, 60.8% of genes; *P* = 1.47 × 10^−3^) and/or for the TF-complex Rb:E2F-1:DP-1 (motif ‘TTTSGCGC’, 34.3%, P = 1.26 × 10^−3^). The E2F recognition sites are found in the promoters of genes involved in cell cycle or DNA replication and a specific role of E2F has been demonstrated in regulating mammalian endocycle, yielding in highly polyploid cells such as placental syncytiotrophoblasts^34^. A recent study showed that placental transcriptional network is tightly coordinated by activation and repression through the two distinct arms of the E2F TF-family and this mechanism has direct implications for extra-embryonic cell proliferation, placental development and fetal viability^35^.

## Discussion

Recurrent pregnancy loss (RPL) represents an insufficiently acknowledged common disease without adequate management options. Research on the causes and mechanisms of RPL represent a challenge, as it is a complex disorder involving both partners and their miscarried pregnancies^36^. There are worldwide increasing trends in the prevalence of RPL due to growing age of couples aiming at childbirth, as well as availability of assisted reproductive technologies (ART), such as *in vitro* fertilization (IVF) and Intracytoplasmic Sperm Injection (ICSI). In the current study we sought to determine the placental differential gene expression signature associated with RPL and bring novel insights into the involved biological pathways with the ultimate aim to identify potential candidates for the development of therapies for this complex disorder.

As the first main outcome we report novel molecular mechanisms behind directing a pregnancy towards a miscarriage scenario. Our data suggests that this scenario is programmed initially at the genomic level through winding down DNA replication, chromatin assembly, RNA processing machinery and basic cellular metabolic function – all key processes to guarantee rapid proliferation of trophoblastic cells essential for placental formation and invasion to establish a viable pregnancy. Chorionic villous samples from RPL cases compared to electively terminated, but uncomplicated pregnancies (ETP) had substantially decreased transcript levels of histones, regulatory RNAs (snRNA, snoRNAs) and genes involved in telomere, spliceosome, ribosomal and mitochondrial and intra-cellular signaling functions (Fig. 2a,b,d,e; Fig. 3; Supplementary Data S3). Notably, no such systematic differential expression was detected for the microRNA transcript profile between the RPL and ETP samples (Fig. 2c,f; Supplementary Data S4).

As an important novel insight, the data points to E2F transcription factor (TF) family as a molecular mechanism to trigger the coordinated process in programming the fetal genome to gradually stop the maintenance of basic nuclear and cellular functions. Transcriptional activity of the majority of differentially expressed genes in RPL placentas was predicted to be regulated by E2F TFs or E2F:DP1 heterodimer complexes (Table 2; Supplementary Data S7). In mammals, E2F family is exclusively involved in cell cycle regulation and DNA synthesis, controlling cell-cycle progression from G1 to S phase via the coordinated action activators (E2F1-E2F3) and repressors (e.g. E2F7-E2F8). A specific crucial role of E2F transcription factors has been shown in orchestrating endocycle in mammalian cells – genome replication in the absence of cell division^34^. This is a key feature in implantation process and normally functioning trophoblastic cells, and is represented in the most extreme form in multinuclear syncytiotrophoblast layer in the human placenta and in trophoblastic giant cells in mouse^37^. Consistent with the critical role of endocycle process in the normal placental development and function, our recent study identified an extensive load of somatic structural variants in the human placental genome and the highest fraction of genomic rearrangements was detected in normal pregnancies^38^. Endoreplication is essential for normal early development in many eukaryotic organisms^39^ and the role of E2F transcription factors in the involved processes appears to be evolutionarily conserved among mammals and insects^40^.

In mammals, E2F transcription factors have a key role in coordinating the placental transcriptional network to guarantee proper cellular proliferation, placental development and ultimately, fetal viability^35^,^41^. Murine *E2f7*−/− and *E2f8*−/− embryos exhibited a severely compromised placenta associated with ectopic proliferation, apoptosis, and altered differentiation of extra-embryonic cell lineages and embryonic death by E11.5^35^,^42^. The loss of *Dp1* – acting in unison with E2F forming functional heterodimers, leads to death *in utero* due to failed extra-embryonic development represented by compromised expansion of the ectoplacental cone and chorion, and impaired endoreduplication in trophoblast giant cells^43^. Our data are also consistent with studies, which have implicated E2F transcription factors in regulating the expression of mitochondria-associated genes^44^,^45^. This novel function of E2F factors together with the crucial role in cell cycle has been suggested to have a major impact on cell viability, independent of induction of apoptotic genes.

Importantly, the E2F transcription factor family plays also a critical role in controlling tumor progression, angiogenesis and metastasis in cancer^46^,^47^,^48^. In perspective, knowledge on the mechanisms leading to failed proliferation and invasion of trophoblastic cells and impaired placental development may also have implications to cancer biology and therapeutic solutions.

Seminal microarray-based investigations of transcriptome and proteome of chorionic villi from RPL patients have demonstrated a dysregulated (mostly increased) expression of genes involved in regulating of immune function, apoptosis, angiogenesis and cytotrophoblast invasion^11^,^12^,^49^,^50^,^51^. All these processes are also represented among the upregulated genes in our dataset (Fig. 3A; details and references in Supplementary Data S3). As an important result, our data provided support for the involvement of several previously suggested genes linked to adverse pregnancy outcomes and/or placental function. Among 51 loci with significantly increased transcript levels there were five genes with independent literature evidence for involvement in RPL (*C3, PHLDA2, ATF4, ICAM1, GPX4*). Additional 8 genes are associated with pregnancy complications or placental function (Fig. 3B). Some of these genes are dysregulated in multiple adverse pregnancy complications, such as imprinted and maternally expressed gene *PHLDA2*, which is linked to RPL, fetal growth restriction (FGR) and preeclampsia^20^,^24^,^25^,^26^. A number of pregnancy complications including RPL, FGR, hypertensive disorders in pregnancy and preterm birth are associated with excessive or misdirected complement activation, where *C3* has a distinct role in early pregnancy^18^.

To our knowledge, this is the first study using RNA-Seq to profiling of the transcriptome and miRNome of chorionic villi representing RPL cases in comparison with uncomplicated early pregnancy. Choice of RNA-Seq using ribo-depleted (not polyA-enriched) total RNA for library preparation enabled interrogation of the full profile of placental transcriptome including ncRNAs and histone genes, which are typically not represented on commercial microarrays. As a limitation in our study, the replication experiments differed from the discovery study in initial preparation of samples. For the discovery RNA-Seq and miRNA-Seq experiments we used chorionic villi, which were dissected under stereomicroscope in a specialized laboratory and karyotyped to exclude any gross chromosomal aberration (RPL, n = 2; ETP n = 8). Chorionic villous samples used only in the TaqMan RT-qPCR experiment (RPL, n = 12; ETP, n = 16) were dissected in the delivery room and the karyotype of the fetus was analysed only for some cases. Thus, the placental samples used in the replication experiments may have occasionally included also maternal decidual cells or represent pregnancies with chromosomal anomalies. Despite this limitation, Taqman RT-qPCR replication experiments confirmed a substantial decrease in the expression of *HIST1H1B* and *HIST1A4H* genes (encoding histone 1 and histone 4) in the RPL samples.

We acknowledge that any molecular studies targeting the causes and mechanisms of recurrent pregnancy loss in humans have their limits in terms of sample size and data interpretation, which are already predefined by the complexity of this disorder. The nature of miscarriage process does not allow to design a study capable to distinguish properly, whether the reported altered pathways represent underlying mechanisms causing or triggering pregnancy loss or merely accompanying consequences to the already ‘programmed’ miscarriage scenario. Thus, it is not possible to ultimately exclude that the findings of the current study reflect the processes associated with fetal demise. However, in the latter case we would expect the observed changes to appear prominently on the principal component analysis and be less specific to certain biological pathways.

## Conclusions

The compiled evidence suggests that early pregnancy losses may be triggered and/or mediated by decreased functional capacity of the placental genome. The chorionic villous samples from recurrent pregnancy loss (RPL) cases compared to normal 1^st^ trimester pregnancy had substantially reduced transcript levels of proteins and ncRNAs involved in chromatin assembly, RNA processing, mitochondrial function and other components of the basic nuclear and cellular machinery. We expect that these mechanisms are not unique to RPL cases, but similar processes may accompany sporadic pregnancy loss events. The early placenta is characterized by extremely rapid development and growth, proliferation and invasion of trophoblastic cells that require high and constant supply of all these molecules in order to establish a viable pregnancy.

The current study detected for the first time that majority of the differentially expressed genes in chorionic villi of RPL cases are regulated by E2F family of transcription factors and their functional partner DP1, both known to be involved in endoreplication and placental development. This favors the scenario that pregnancy loss is programmed as a cascade of events, which coordinately directs the developing placenta towards shutting down all the basic nuclear and cellular functions, and finally leading to miscarriage. The role of E2F/DP1 transcription factors and linked pathways in orchestrating this cascade of events needs further investigations not only from the basic research angle, but also as a potential source to identify therapeutic targets for RPL.

The study detected differential expression of a number of genes previously reported in connection with adverse pregnancy outcomes and/or placental function, including five genes implicated in RPL (*C3, PHLDA2, ATF4, ICAM1, GPX4*). The list of differentially expressed genes in RPL represents a source for generating mouse models to investigate *in vivo* treatment options for RPL, for screening genetic variants predisposing to pregnancy failure and for developing biomarkers for monitoring of early pregnancies in clinical risk cases (e.g. pregnancies achieved through ART).

## Methods

### Ethics statement

The study was approved by the Ethics Review Committee of Human Research of the University of Tartu, Estonia (permissions no 117/9, 16.06.2003; 146/18, 27.02.2006; 150/33, 19.06.2006; 212/M-32, 09.03.2012) and it was carried out in compliance with the Helsinki Declaration. A written informed consent to participate in the study was obtained from each individual prior to recruitment. All study participants were recruited and the study material was collected at the Women’s Clinic of Tartu University Hospital, Estonia in 2003–2012. All participants were of white European ancestry and living in Estonia. All methods were carried out in accordance with approved guidelines.

### Study patients and placental samples of RPL and ETP groups

Recurrent pregnancy loss (RPL) patients analyzed in the current study (in total n = 14; maternal age 32.9 ± 4.6 years, gestational age 58.9 ± 17.3 days) had experienced at least two consecutive miscarriage events before this case. The following known strong risk factors of pregnancy loss had been excluded: abnormal menstrual cycle, genital infections, antiphospholipid syndrome, thrombophilic mutations in female partner and abnormal karyotype of both partners of the RPL couples (details in **Supplementary Methods**). The two women with the index RPL pregnancies, allocated to RNA-Seq/miRNA-Seq analysis of chorionic villi, had experienced five (RPL1) and six (RPL2) clinically confirmed pregnancy losses before the index case. The RPL placental samples were collected at surgical removal of conceptus shortly (within 24 hours) after detection of fetal death. In both cases, the most recent ultrasound scan confirming the heart beats and normal growth of the fetus had been performed five (RPL1) and seven (RPL2) days before the event. Gestational age in used the study for RPL cases was based on the measurement of crown-rump-length of the fetus.

The patients in ‘elective termination of pregnancy’ group (ETP; in total n = 32) had experienced normal 1^st^ trimester pregnancies with no maternal or fetal clinical complications until the termination of the pregnancy (maternal age 26.8 ± 6.2 years, gestational age 64.5 ± 13.2 days). None of the patients had experienced any clinically confirmed pregnancy losses in their reproductive history.

For the RNA-Seq/miRNA-Seq profiling, the tissue samples were obtained immediately after surgical termination of pregnancy (ETP group, n = 8) or surgical removal of conceptus (RPL group; n = 2) under general anaesthesia (Table 1). The maternal tissue was removed under a stereomicroscope (Discovery V8, Zeiss) and chorionic villi containing both cyto- and syncytiotrophoblast cells were placed into RNAlater solution (Ambion Inc, Life Technologies) and stored at −80 °C without any further manipulation. For all samples entering RNA-Seq/miRNA-Seq, part of the purified trophoblast cell population was karyotyped and it confirmed normal male or female karyotype in all cases (United Laboratories, Tartu University Hospital). Both RPL samples were subjected in parallel to RNA-Seq and miRNA-Seq dataset generation, whereas for the ETP samples the remaining clinical material after RNA-Seq experiment enabled to perform miRNA-Seq for 5/8 ETP samples.

Taqman RT-qPCR validation experiments included an extended placental sampleset from the ETP (n = 24) and RPL (n = 12) groups (Supplementary Table S1). These placental samples had not been subjected to dissection of chorionic villi under microscope. After washing in PBS to remove blood, chorionic villi were separated visually, followed by snap-freezing in liquid nitrogen or placing into RNAlater solution. Samples were kept at −80 °C until RNA isolation^52^ (details in **Supplementary Methods**).

### RNA-Seq and miRNA-Seq

Total RNA was extracted from 200–300 mg of homogenized placental tissue using TRIzol reagent (Invitrogen, Life Technologies). RNA intergrity number (RIN) values for the two RPL samples were 7.8 and 7.9 and for the eight ETP samples ranged from 6.2 to 8.9 (median 7.9).

To produce RNA-Seq libraries total RNA was further purified with RNeasy MinElute columns (Qiagen, Netherlands) according to the manufacturers’ protocol. High quality DNA-free total RNA (5 μg) was used for rRNA depletion (Ribo-Zero™ rRNA Removal Kit, Epicentre) and library preparation with cDNA synthesis (Life Technologies) followed by Nextera™ Technology (Illumina). For miRNA-Seq, initial small-RNA libraries were prepared from 1 μg total RNA (TruSeq Small RNA kit, Illumina), followed by microRNA enrichment (Caliper LabChipXT, PerkinElmer) according to manufacturer’s protocols. RNA-Seq (46 bp paired end reads for ETP samples; for RPL samples 101 bp paired end reads were trimmed to 50 bp) and miRNA-Seq libraries were sequenced on Illumina HiSeq 2000.

Initial RNA-Seq data analysis and preparation was conducted by the RNA-Seq pipeline v2.4 (Sequencing Unit, FIMM Technology Centre) consisting of FastQC version 0.10.0 for quality control; reads were filtered for adaptor, rRNA and mtDNA sequences as well as homopolymer stretches using custom python scripts. Reads were aligned to human genome assembly (GRCh37.p7/hg19) with TopHat version 2.0.3^53^. Transcript quantification was conducted with Cufflinks v 2.0.2^54^ with reference annotation (measured as FPKM) and gene expression was quantified by htseq-count^55^ (as raw read counts). Further details of library preparation, RNA-Seq and basic bioinformatics of raw data is provided in^14^.

Initial analysis of microRNA sequencing data included removal of the 3′ adaptor sequence and discarding reads with the trimmed length < 14 bp. Reads were mapped with bowtie (v0.12.7)^56^ to the reference genome (GRCh37.p7/hg19) allowing a maximum of 1 mismatch. Calculation of the raw read counts was performed using a modified version of *Emir*^57^ bioinformatic pipeline using mature miRNA expression database (mirBASE v18).

### RNA-Seq dataset for term placental samples from normal pregnancy

The RNA-Seq dataset representing term placental transcriptome in normal, uncomplicated pregnancies (group ‘Term norm’) was derived from previous published report^14^ and utilized as a reference to compare with the 1^st^ trimester placental gene expression (see **Supplementary Methods**).

### Taqman RT-qPCR

Five genes were selected for confirmation of differential expression using Taqman RT-qPCR method and in an independent sample-set. The inclusion criteria were genes with high fold change (>4), substantial placental gene expression (>150 normalized read count) and commercially available assays from Applied Biosystems (Life Technologies; Supplementary Table S2). Among the down-regulated genes we selected for validation experiments *HIST1H1B* (encoding histone H1) and *HIST1H4A* (histone H4), and among the upregulated gene-list *C3* (complement component 3), *CAPNS1* (calpain, small subunit 1) and *PLTP* (phospholipid transfer protein). Among these, *C3* and *PLTP* have also literature evidence for the involvement of RPL and/or function at the feto-maternal interface^18^,^58^,^59^. For *CAPNS1* a role in acute allograft rejection has been reported^60^.

Gene expression was quantitated by biplex RT-qPCR of the target gene and housekeeping gene *YHWAZ* sequence using pre-made TaqMan Gene Expression Assays (Hs03044281_g1, Applied Biosystems, Life Technologies). *YHWAZ* was selected as the most stable ready-to-use housekeeping gene based on data from RNA sequencing and literature sources^61^. cDNA was synthesized from 1 μg total RNA according to the manufacturer’s instructions (SuperScript III Reverse Transcriptase, Life Technologies). All qPCR reactions were performed in triplicate in 384 micro-well plates in ABI 7900HT Real-time PCR system (Applied Biosystems, Life Technologies) using HOT FIREPol^®^ Probe qPCR Mix (Solis BioDyne, Tartu, Estonia). Full experimental details of the Taqman RT-qPCR are provided in **Supplementary Methods**.

### Statistical analysis

Differential expression in RNA-Seq and miRNA-Seq data was tested using DESeq^62^ and DESeq2^63^ packages for R^64^. Read counts from htseq-count (for RNA-Seq) and Emir pipeline (for miRNA-Seq) were used as input and built-in normalization algorithms of DESeq and DESeq2 were used. Outlier detection and handling was performed using the default method in DESeq. In DESeq2 outliers were replaced using the *replaceOutliersWithTrimmedMean* function with default Cook’s distance cutoff. In the current study a gene was considered as differentially expressed, when the statistical tests simultaneously satisfied the following thresholds: FDR adjusted *P* < 0.1 for DESeq and FDR adjusted *P* < 0.05 for DESeq2. In RNA-Seq and miRNA-Seq datasets, genes with mean normalized expression < 50 reads in all samples (n = 39,109 RNA-Seq; n = 555 miRNA-Seq) were considered transcriptional noise and filtered out from the analysis. Statistical testing, principal component analysis (PCA) and hierarchical clustering (Pearson correlation as the distance function) were performed in R.

Gene set enrichment analysis for the differentially expressed genes in RPL (n = 2) compared to ETP (n = 8) chorionic villous samples was performed using g:Profiler^65^. Enrichment was tested for the functional categories defined in Gene Ontology (GO), Reactome (REAC) databases, as well as for the transcription binding site motifs from TRANSFAC database and miRBase microRNAs. Functional profiling applied the default input option ‘Only annotated genes’ (n = 83 independent genes from Supplementary Data S3) and selected output option ‘Best per parent group (strong)’. The analysis criteria to claim statistical significance applied g:SCS threshold as recommended by the software developer. g:SCS threshold is a value pre-calculated for query list sizes up to 1000 genes. The algorithm considers the set structure underlying gene sets annotated to terms of each organism, and should therefore give a tighter threshold to significant results compared to Bonferroni correction or Benjamini-Hochberg FDR.

Statistical analyses for Taqman RT-qPCR results were performed using statistical package STATA version 13.1. Significance of RT-qPCR measurements among the study groups was assessed by Wilcoxon test (no covariates) and logistic regression (gestational age as covariate). Results with nominal *P*-values *P* < 0.01 were considered as significant. Additional details of the statistical analysis are provided in **Supplementary Methods**.

## Additional Information

**How to cite this article**: Sõber, S. *et al*. RNA sequencing of chorionic villi from recurrent pregnancy loss patients reveals impaired function of basic nuclear and cellular machinery. *Sci. Rep.* **6**, 38439; doi: 10.1038/srep38439 (2016).

**Publisher's note:** Springer Nature remains neutral with regard to jurisdictional claims in published maps and institutional affiliations.

## Supplementary Material

Supplementary Information

Supplementary Dataset 1

Supplementary Dataset 2

Supplementary Dataset 3

Supplementary Dataset 4

Supplementary Dataset 5

Supplementary Dataset 6

Supplementary Dataset 7

## Figures and Tables

**Table 1:** Samples utilized for placental transcriptome sequencing and clinical characteristics of respective pregnancies.

	ETP controls	RPL cases	Term norm
No of samples	8	2	8
Type of samples	1^st^ trimester chorionic villi	1^st^ trimester chorionic villi	3^rd^ trimester placenta
Maternal age (years)	25.5 [18–33]	35.5 [32, 39]	33 [18–37]
Paternal age (years)	28.5 [20–47]	33 [30, 36]	34 [22–38]
Pre-pregnancy BMI (kg/m^2^)	22.2 [19.4–29.7]	20.3 [19.9, 20.6]	23.8 [17.4–30.0]
Nulliparity (n, %)	2 (25%)	1 (50%)	3 (38%)
Gravidity	2.5 [1–5]	7.5 [7, 8]	2 [1–5]
Previous pregnancy losses	0	5.5 [5, 6]	0 [0–1]
Previous ETP	1 [0–3]	0.5 [0, 1]	0 [0–1]
Gestational age at sampling (days)	60 [51–81]	55.5 [44, 67]	284 [268–291]
Fetal sex (female/male)	5/3	1/1	3/5
Newborn weight (g)	N/A	N/A	3756 [3102–4220]

Data are given as median and range, except where indicated differently; nulliparity refers to no previous childbirth; gravidity refers to total number of experienced pregnancies.

BMI, body mass index; ETP, elective termination of pregnancy; Term norm, placental samples representing uncomplicated normal pregnancy at term; RPL, recurrent pregnancy loss; n, number; N/A, not applicable.

**Table 2:** Overrepresented functional categories among the differentially expressed genes in placental chorionic villi in RPL compared to ETP samples.

Significantly overrepresented functional categories			1Enrichment analysis			Gene expression levels in RPL compared to ETP	
type	ID	Name	Pathway genes (%)	Query genes (%)	Corrected *P*-value	Increased	Decreased
A. Gene Ontology (GO)							
CC	0000786	nucleosome	32.1	41.0	2.34 × 10^−53^	none	histones (n = 34)
BP	0006334	nucleosome assembly	18.8	32.5	3.27 × 10^−34^	none	histones (n = 27)
MF	0046982	protein heterodimerization activity	6.4	37.3	5.07 × 10^−25^	*ATF4*	histones (n = 31)
CC	0005576	extracellular region	1.2	67.5	7.36 × 10^−14^	*CD74, CTSA, FTL,ICAM1, PLTP, MAPK3, PPP2CB, CDC37, SYNGR2, MDK, RAB35, C3, CAPNS1, NBL1, FRZB, GPX4, LRG1, FAM20C, LAMB3*	histones (n = 31)Mitochondrial function: *MT-RNR2, MTRNR2L9, MTRNR2L8, MTRNR2L10, MTRNR2L3, MTRNR2L1*
BP	0035574	histone H4-K20 demethylation	43.8	8.4	6.46 × 10^−10^	none	histones (n = 7)
MF	0003677	DNA binding	1.5	45.8	6.92 × 10^−10^	*EGR1, ZFP36, ATF4, WBP2*	histones (n = 34)
BP	0006303	double-strand break repair via non-homologous end joining	10.4	8.4	4.11 × 10^−5^	none	histones (n = 7)
BP	0007264	small GTPase mediated signal transduction	1.9	21.7	2.29 × 10^−4^	*MAPK3, PPP2CB, RAB35, ARL4C*	histones (n = 14)
B. TRANScription database (TRANSFAC)							
tf	M00919_1	E2F; motif: NCSCGCSAAAN	3	14.7	5.31 × 10^−6^	*CAPNS1, WBP2, ARL4C*	histones (n=12)
tf	M00516_0	E2F; motif: TTTSGCGCGMNR	3.1	13.7	1.33 × 10^−5^	*ARL4C*	histones (n = 13)
tf	M00939_1	E2F; motif: TTTSGCGSG	2.6	14.7	3.34 × 10^−5^	*CAPNS1, WBP2, ARL4C*	histones (n = 12)
tf	M00918_1	E2F; motif: TTTSGCGSG	2.8	12.7	1.18 × 10^−4^	*CAPNS1, WBP2, ARL4C*	histones (n = 10)
tf	M00738_0	E2F-4: DP-1; motif: TTTSGCGC	1.1	30.4	4.01 × 10^−4^	*CDC37, PDLIM1,ZFP36, WBP2, TMUB1,ARL4C*	histones (n = 11)*SNORA*-s (n = 4)*RNU* genes (n=2)*HNRPA0, RN7SK*
tf	M00920_1	E2F; motif: NKCGCGCSAAAN	2.4	12.7	8.39 × 10^−4^	*WBP2, ARL4C*	histones (n = 11)
tf	M00209_0	NF-Y; motif: NCTGATTGGYTASY	1.5	20.6	9.53 × 10^−4^	*PI4K2A, C1ORF122*	histones (n = 18), *SNORA38B*
tf	M00210_0	OCT-x; motif: CTNATTTGCATAY	1.3	22.5	9.53 × 10^−4^	*EGR1*	histones (n = 19)*RNU* genes (n = 3)
tf	M00740_0	Rb:E2F-1:DP-1; motif: TTTSGCGC	1	34.3	1.26 × 10^−3^	*CDC37, PDLIM1,ZFP36, WBP2, ARL4C,TMUB1*	histones (n = 20)*SNORA*-s (n = 4)*RNU* genes (n = 2)*HNRPA0, RN7SK, MTRNR2L10*
tf	M00428_0	E2F-1; motif: NKTSSCGC	0.7	60.8	1.47 × 10^−3^	*CD74, CTSA, ICAM1,PDLIM1, SYNGR2, MDK, RAB35, EGR1, PIGT,ZFP36, ATF4, NINJ1,WBP2, SLC16A2,PI4K2A, TMUB1, GPX4,PRELID1, FAM20C, GRINA, PHLDA2, ARL4C*	histones (n = 30)*SNORA*-s (n = 2) *RNU* genes (n = 4)*HNRPA0, SCARNA5,SSTR5-AS1,MTRNR2L10*

^1^Gene enrichment analysis was implemented in g:Profiler software^65^. The query included differentially expressed genes from Supplementary Data S3, which were annotated in ENSEMBL. The analysis used a conservative output function ‘Best per parent group (strong)’. Overrepresented functional categories in GO and TRANSFAC databases with highly significant statistical support are shown (*P* < 1 × 10^–2^). Full analysis details and gene enrichment profiling for the REACtome categories are provided in Supplementary Data S7. Query to miRBase database did not result in any statistically significant outcome.

BP, Biological Process; CC, Cellular Component; ETP, electively terminated pregnancy with no gestational complications; MF, Molecular Function; RPL, recurrent pregnancy loss; tf, transcription factor binding motif.
