# Supplementary material for: RNA sequencing of chorionic villi from recurrent pregnancy loss patients reveals impaired function of basic nuclear and cellular machinery
Source: Sci Rep. 2016 Dec 8;6:38439. doi: 10.1038/srep38439 (PMC5143936; doi:10.1038/srep38439)
Supplement: Supplementary Information [file srep38439-s1.pdf]

Supplementary Information for

Siim Sõber, Kristiina Rull, Mario Reiman, Piret Ilisson, Pirkko Mattila, Maris Laan

'RNA sequencing of chorionic villi from recurrent pregnancy loss patients reveals impaired function of basic nuclear and cellular machinery'

List of Supplementary Material:

Supplementary Methods, Text S1 Full details of samples, materials and methods

Supplementary Figure S1. RNA-Seq and small RNA-Seq metrics for the analyzed placental tissue samples.

Supplementary Figure S2. High correlation between the transcriptomes of recurrent pregnancy loss placental samples.

Supplementary Figure S3. Taqman RT-qPCR analysis of differential expression of *C3, PLTP* and *CAPNS1* genes in the placental tissues representing recurrent pregnancy loss and electively terminated uncomplicated pregnancies

Supplementary Table S1. Samples utilized for Taqman RT-qPCR and clinical characteristics of ETP and RPL samples

Supplementary Table S2. Genes selected for TaqMan RTqPCR and their Assay IDs.

Supplementary Data S1 Top 200 transcripts with the highest measured gene expression in FPKM in the chorionic villous samples from 1st trimester electively terminated pregnancies (ETP) and recurrent pregnancy loss (RPL).

(a separate .xls file, 78 kb)

Supplementary Data S2. Mean DESeq2 normalized read counts for all detected microRNAs (n=867) in placental samples from electively terminated pregnancies (ETP), recurrent pregnancy loss (RPL) and normal term deliveries (Term norm).

(a separate .xls file, 131 kb)

Supplementary Data S3. Differentially expressed genes between chorionic villous samples representing 1st trimester ETP and RPL.

(a separate .xls file, 108 kb)

Supplementary Data S4. Results for differential expression tests between miRNA-seq datasets chorionic villous samples representing 1st trimester ETP and RPL.

(a separate .xls file, 75 kb)

Supplementary Data S5. Impact of individual analysed recurrent pregnancy loss samples (RPL1, RPL2) on the differential expression outcome.

(a separate .xls file, 187 kb)

Supplementary Data S6. Top 200 genes from the analysis of maternal age dependent differential expression in ETP (elective termination of otherwise normal pregnancy) samples.

(a separate .xls file, 808 kb)

Supplementary Data S7. Overrepresented functional categories among the differentially expressed genes in placental chorionic villi in RPL compared to ETP samples

(a separate .xls file, 39 kb)

Supplementary Methods, Text S1 Full details of samples, materials and methods

First trimester placental samples and detailed description of study subjects

Elective (surgical) termination of pregnancy (ETP group): The subjects (n=8 in the discovery RNA-Seq study; n=24 for TaqMan RT-qPCR experiment) had experienced normal first trimester pregnancies with no maternal or fetal clinical complications until the elective termination of the pregnancy (mean maternal age 25.5±5.9 years, and 27.3±6.4 respectively; gestational age 64.6±13.9 days and 64.6±11.6 days respectively; Table 1 and Supplementary Table S1). None of the patients had experienced any clinically confirmed pregnancy losses in their reproductive history.

Recurrent pregnancy loss (RPL group): Discovery analysis was performed on chorionic villous samples dissected under stereomicroscope from two RPL cases (maternal age 32 and 39 years, gestational age 44 and 67 days). Taqman RT-qPCR experiments included the two discovery samples, as well as 12 additional RPL placental samples (visual separation of chorionic villi; mean maternal age 32.5±4.6 years, gestational age 59.4±18.1 days; Table 1; Supplementary Table S1). All recruited RPL patients had experienced at least two consecutive miscarriage events before the index case. The following known contributing factors of pregnancy loss had been excluded in RPL cases: abnormal menstrual cycle (<21 days and >35 days), genital infections, antiphospholipid syndrome (positive anti-cardiolipin and/or β2 glycoprotein 1 antibodies), thrombophilic mutations (*V* Leiden c.6191G>A, p.Arg506Gln; *Coagulation II* c.20210G>A) in female partner and abnormal karyotype of either of the partners of the RPL couples. No anticoagulant therapy (low molecular weight heparin, acetylsalicylic acid) was applied during the pregnancy. Four patients had been prescribed dydrogesterone or progesterone at variable gestational ages, with variable treatment period and dosage per day. Due to low number of involved patients and multiplicity of treatment schemes, the effect of medication on mRNA expression was not assessed.

The RPL cases recruited to the placental RNA-Seq/miRNA-seq discovery study had experienced five (RPL1) and six (RPL2) clinically confirmed pregnancy losses before the index case. The RPL placental

samples were collected at surgical removal of conceptus shortly (within 24 h) after detection of fetal death. In both cases, the most recent ultrasound scan confirming the heart beats and normal growth of the fetus had been performed five (RPL1) and seven (RPL2) days before the event. In both patients diagnostic hysteroscopy, performed after index pregnancy revealed partial (up to 1 cm) uterine septum that was removed during the procedure. The anomaly has not been detected at multiple U/S performed during previous years before the index pregnancy. After the index pregnancy and removal of uterine septum, both patients have had one term delivery and further pregnancy losses.

All 1st trimester placental tissue samples were obtained immediately after surgical termination of pregnancy (ETP group) or surgical removal of conceptus (RPL group) under general anaesthesia. The placental material was washed in solution containing 15 ml Dulbecco's Phosphate Buffered Saline (DPBS), 0.3 ml penicillin (10000U/ml) - streptomycin 10000µg/ml) solution and 2 drops of heparine (5000U/ml) to remove maternal blood. Removal of the maternal tissue was performed under a stereomicroscope (Discovery V8, Zeiss). For the RNA-Seq/miRNA-seq experiments, the chorionic tissue was further placed into DPBS with no additives. Chorionic villi containing both cyto- and syncytiotrophoblast cells were placed into RNAlater solution (Ambion Inc, Life Technologies) and stored at -80°C without any further manipulation (cell sorting, culturing). For all samples entering RNA-Seq/miRNA-seq, part of the purified trophoblast cell population was karyotyped and it confirmed normal male or female karyotype in all cases (United Laboratories, Tartu University Hospital). Both RPL samples were subjected in parallel to RNA-Seq and miRNA-seq dataset generation, whereas for the ETP samples the remaining clinical material after RNA-Seq experiment enabled to perform miRNA-seq for 5/8 ETP samples.

Taqman RT-qPCR experiments included additional placental samples (ETP n=24; RPL n=12). For these samples, the washing step in PBS was followed by immediate visual separation of chorionic villi, which were snap-frozen in liquid nitrogen or placed into RNAlater solution (Ambion Inc, Life Technologies) and kept at -80°C until RNA isolation [1].

Placental samples of normal term pregnancies

The RNA-Seq dataset representing term placental transcriptome in normal, uncomplicated pregnancies (Term norm) was derived from previous published report [2] and utilized as a reference to compare with the 1st trimester placental gene expression. miRNA-seq data of the identical samples (n=8) was generated in the current study.

Term placental samples representing normal pregnancy originate from the REPROgrammed fetal and/or maternal METAbolism (REPROMETA) sample collection [2–4]. The study group in the current study (n=8, Table 1) was initially reported in our recent study comprised of uncomplicated term pregnancies (maternal age 29.3±7.9, gestational age 278.6±11.5), which resulted in the delivery of a newborn with normal birth weight for its gestational age (birth-weight between 10th-90th centile, [5]). Cases with documented fetal anomalies, chromosomal abnormalities, families with history of inherited diseases and patients with known pre-existing diabetes mellitus, chronic hypertension and chronic renal disease were excluded. Placentas (stored at +4°C) were sampled within 1 h after vaginal delivery (n=5) or elective caesarean section without labor (n=3). Full-thickness blocks of 2-3 cm were taken from the middle region of the placenta. Collected tissue samples were washed with 1x PBS to remove contamination of maternal blood, placed immediately into RNAlater solution (Ambion Inc, Life Technologies) and kept at -80°C until RNA isolation.

RNA extraction and purification

Total RNA was extracted from 200-300 mg of homogenized placental tissue using TRIzol reagent (Invitrogen, Life Technologies). For RNA-Seq protocol, it was further purified with RNeasy MinElute columns (Qiagen, Netherlands) and for Taqman RT-qPCR applications with NucleoSpin® II Isolation Kit (Macherey-Nagel GmbH & Co. KG, Düren, Germany) according to the manufacturers' protocols. Purity level and concentration of isolated total RNA was measured using NanoDrop® ND-1000 UV-Vis spectrophotometer (Thermo Fisher Scientific Inc., USA) and RIN (RNA integrity number) was estimated using Agilent 2100 Bioanalyzer (Agilent Technologies, USA).

Library preparation and basic bioinformatics of raw data for RNA-Seq

Preparation of RNA-Seq sequencing libraries, sequencing of transcriptomes and basic bioinformatic processing of the raw sequencing data (quality control, read alignment and transcript and gene expression estimation) were performed at the Sequencing Unit of Finnish institute of Molecular Medicine (FIMM), University of Helsinki, Finland. High quality DNA-free total RNA (5 μg) was used for depletion of ribosomal RNA (Ribo-Zero™ rRNA Removal Kit, Epicentre, Madison, WI, USA). The rRNA depleted RNA was purified (NucleoSpin® RNA Clean-up XS, Macherey-Nagel, Duren, Germany) and reverse transcribed to double-stranded cDNA (SuperScript™ Double-Stranded cDNA Synthesis Kit, Life Technologies, Carlsbad, CA, USA). Random hexamers (New England BioLabs, Ipswich, MA, USA) were used for priming the first strand synthesis reaction and SPRI beads (Agencourt AMPure XP, Beckman Coulter, Brea, CA, USA) for purification of cDNA.

Nextera™ Technology (Illumina, San Diego, CA, USA) was used for preparation of RNA-Seq libraries. In order to add the Illumina specific bridgePCR compatible sites as well as bar codes and enrich the library, limited-cycle PCR (5 cycles) was done according to instructions of Nextera system. SPRI beads were used for purification of the PCR-products and the library QC was evaluated by Agilent Bioanalyzer (Agilent Technologies, Santa Clara, CA, USA). C-Bot (TruSeq PE Cluster Kit v3, Illumina, San Diego, CA, USA) was used for cluster generation and Illumina HiSeq2000 platform (HiSeq TruSeq v3 reagent kit) for paired end sequencing with 2 x 46 bp read length for ETP samples and 2 x 101 bp read length for RPL samples. Reads from RPL samples were trimmed from the 3' end to 50 bp to match the ETP samples.

Initial data analysis and preparation was conducted by the RNA-Seq pipeline v2.4 (FIMM) consisting of FastQC version 0.10.0 (S. Andrews. FastQC (2011) http://www.bioinformatics.babraham.ac.uk/projects/fastqc/, date of access: 29/06/2015) for quality control; reads were filtered for adaptor, rRNA and mtDNA sequences as well as homopolymer stretches using custom python scripts; read alignment was performed with TopHat version 2.0.3 [6] using bowtie

version 0.12.7 [7]; transcript quantification was conducted with Cufflinks v 2.0.2 [8] with reference annotation (measured as FPKM) and gene expression was quantified by htseq-count [9] (as raw read counts). Human genome assembly (GRCh37.p7/hg19) from Ensembl v67 was used as a reference. Read alignment to genomic regions was estimated with Picard v1.63 (http://picard.sourceforge.net).

Library preparation and basic bioinformatics of raw data for miRNA-seq:

Initial small-RNA libraries were prepared from 1 μg total RNA (TruSeq Small RNA kit, Illumina), followed by microRNA enrichment (Caliper LabChipXT, PerkinElmer) from the 147 bp+/-5% PCR amplification fraction according to manufacturer's protocols. Libraries were sequenced on Illumina HiSeq2000.

Initial analysis of microRNA sequencing data included trimming of reads by removing the 3' adaptor sequence and discarding reads with the trimmed length <14 bp, mapping the remaining reads with bowtie (v0.12.7) to the reference genome (GRCh37.p7/hg19) allowing a maximum of 1 mismatch. Calculation of the raw read counts and normalized RPKM was performed using a modified version of *Emir* bioinformatic pipeline [10], allowing reads that map to genome multiple times (maximum 20) and using mature miRNA expression database (mirBASE v18).

Statistical analysis

Differential expression in RNA-Seq and miRNA-seq data was tested using DESeq [11] and DESeq2 [12] packages for R [13]. Read counts from htseq-count were used as input (number of Ensembl v67 genes with read counts n = 53,893). Genes with mean normalized expression < 50 reads in all samples (n = 39,109 RNA-Seq; n = 555 miRNA-seq) were considered as transcriptional noise and filtered out from the analysis. After the exclusion of genes with negligible placental transcript counts, 14,784 genes from RNA-Seq and 312 miRNAs entered differential expression testing. Built-in normalization algorithms of DESeq and DESeq2 were used. Outlier detection and handling was performed using the default method in DESeq. In DESeq2 outliers were replaced using the *replaceOutliersWithTrimmedMean* function with

default Cook's distance cutoff. Statistical testing indicated that the two software packages, DESeq and DESeq2 differ substantially for their sensitivity in assessment of differential expression. Compared to the seminal DESeq package, analysis with the more recently developed DESeq2 programme produced a markedly higher number of significant results for all conducted differential expression tests with our data. Thus, in the current study a more stringent level of significance was imposed on the test results of DESeq2. A gene was considered as differentially expressed, when the statistical tests simultaneously satisfied the following thresholds: FDR<0.1 for DESeq and FDR<0.05 for DESeq2.

Taqman RT-qPCR experiments and statistical analysis of RT-qPCR data

For an independent experimental validation, gene expression of five protein-encoding genes was analyzed using Taqman RT-qPCR gene expression assays (Applied Biosystems, Life Technologies; Supplementary Table S2). The assessed genes included the top listed genes with high fold change (> 4), substantial gene placental expression (> 150 normalized read count) and commercially available assays from Applied Biosystems, Life Technologies (Supplementary Data S3). Among the down-regulated genes we analyzed the expression level of histone encoding *HIST1H1B* and *HIST1H4A*. From the upregulated gene-list we selected for confirmation genes with previous literature evidence for the involvement of RPL and/or enriched placental expression: *C3 (*complement component 3)*; CAPNS1 (*calpain, small subunit 1); *PLTP* (phospholipid transfer protein).

Gene expression was quantitated by biplex RT-qPCR of the target gene and housekeeping gene *YHWAZ* sequence using pre-made TaqMan Gene Expression Assays (Hs03044281_g1, Applied Biosystems, Life Technologies). *YHWAZ* was selected as the most stable ready-to-use housekeeping gene based on data from RNA-Seq and literature sources [14, 15]. cDNA was synthesized from 1 µg total RNA according to the manufacturer's instructions (SuperScript III Reverse Transcriptase, Life Technologies). All qPCR reactions were performed in triplicate in 384 micro-well plates in ABI 7900HT Real-time PCR system (Applied Biosystems, Life Technologies) using HOT FIREPol® Probe qPCR Mix (Solis BioDyne, Tartu, Estonia) and TaqMan Gene Expression Assays. Negative controls contained either RNA that was

not reverse transcribed or lacked template inputs. RT-qPCR reactions were initially denatured at 95°C for 15 min, followed by 40 cycles of 15 s at 95°C and 1 min at 60°C. Amplification efficiency of TaqMan assays was 90-110%. Relative mRNA expression values were determined by comparative CT method as described previously (Applied Biosystems, Life Technologies).

The statistical analyses for RT-qPCR results were performed using statistical package STATA version 13.1. Fold change (fc) was calculated using median value of expression level of particular transcript in control (ETP) and patient (RPL) groups. Fold change indicates the how much the expression of particular gene is up- (>1) or down-regulated (<1) in cases of RPL compared to normal pregnancies. Significance of RT-qPCR measurements among the study groups was assessed by Wilcoxon test (no covariates) and logistic regression (gestational age as a covariate). For the 5 analyzed genes, the Bonferroni significance level was estimated $\alpha=0.05/5$. Results with $P<0.01$ were considered as significant.

References for Supplementary Methods

1. Rull, K. *et al*. Increased placental expression and maternal serum levels of apoptosis-inducing TRAIL in recurrent miscarriage. *Placenta*, **34**, 141–148 (2013).

2. Sõber, S. *et al.* Extensive shift in placental transcriptome profile in preeclampsia and placental origin of adverse pregnancy outcomes. *Sci. Rep.* **5,** 13336 (2015).

3. Männik, J., Vaas, P., Rull, K., Teesalu, P., Rebane, T. & Laan, M. Differential expression profile of Growth Hormone/Chorionic Somatomammotropin genes in placenta of small- and large-for-gestational-age newborns. *J. Clin. Endocrinol. Metab.* **95**, 2433–2442 (2010).

4. Uusküla, L. *et al*. Mid-Gestational Gene Expression Profile in Placenta and Link to Pregnancy Complications. *PLoS One* **7**:e49248 (2012).

5. Sildver, K.; Veerus, P. & Lang, K. Birth weight percentiles and s associated with birth weight: a registry-based study in Estonia (Sünnikaalukõvarad Eestisja sünnikaalu mõjutavad tegurid:regisripõhine uuring) [in Estonian]. *Eesti Arst* **94,** 465–470 (2015).

6. Kim, D., Pertea, G., Trapnell, C., Pimentel, H., Kelley, R. & Salzberg, S.L. TopHat2: accurate alignment of transcriptomes in the presence of insertions, deletions and gene fusions. *Genome Biol*. **14**, R36 (2013).

7. Langmead, B., Trapnell, C., Pop, M. &Salzberg, S.L. Ultrafast and memory-efficient alignment of short DNA sequences to the human genome. *Genome Biol.* **10**, R25 (2009).

8. Trapnell, C. *et al*. Transcript assembly and quantification by RNA-Seq reveals unannotated transcripts and isoform switching during cell differentiation. *Nat. Biotechnol.* **28**, 511–515 (2010).

9. Anders, S., Pyl, P.T. & Huber, W. *HTSeq A Python Framework to Work with High-Throughput Sequencing Data*. 2014.

10. Buermans, H.P.J., Ariyurek, Y., van Ommen, G. & den Dunnen, J.T., 't Hoen PAC: New methods for next generation sequencing based microRNA expression profiling. *BMC Genomics* **11**, 716 (2010).

11. Anders, S. & Huber W. Differential expression analysis for sequence count data. *Genome Biol*. **11**, R106 (2010).

12. Love, M.I., Huber, W. & Anders, S. Moderated estimation of fold change and dispersion for RNA-seq data with DESeq2. *Genome Biol*. **15**, 550 (2014).

13. R: A Language and Environment for Statistical Computing [http://www.r-project.org]

14. Meller, M., Vadachkoira, S., Luthy, D.A., & Williams, M.A. Evaluation of housekeeping genes in placental comparative expression studies. *Placenta* **26**, 601–607 (2005).

15. Murthi, P., Fitzpatrick, E., Borg, A.J., Donath, S., Brennecke, S.P., & Kalionis, B. GAPDH, 18S rRNA and YWHAZ are Suitable Endogenous Reference Genes for Relative Gene Expression Studies in Placental Tissues from Human Idiopathic Fetal Growth Restriction. *Placenta* **29,** 798–801 (2008).

Supplementary Figure S1 (*see legend on the next page*)

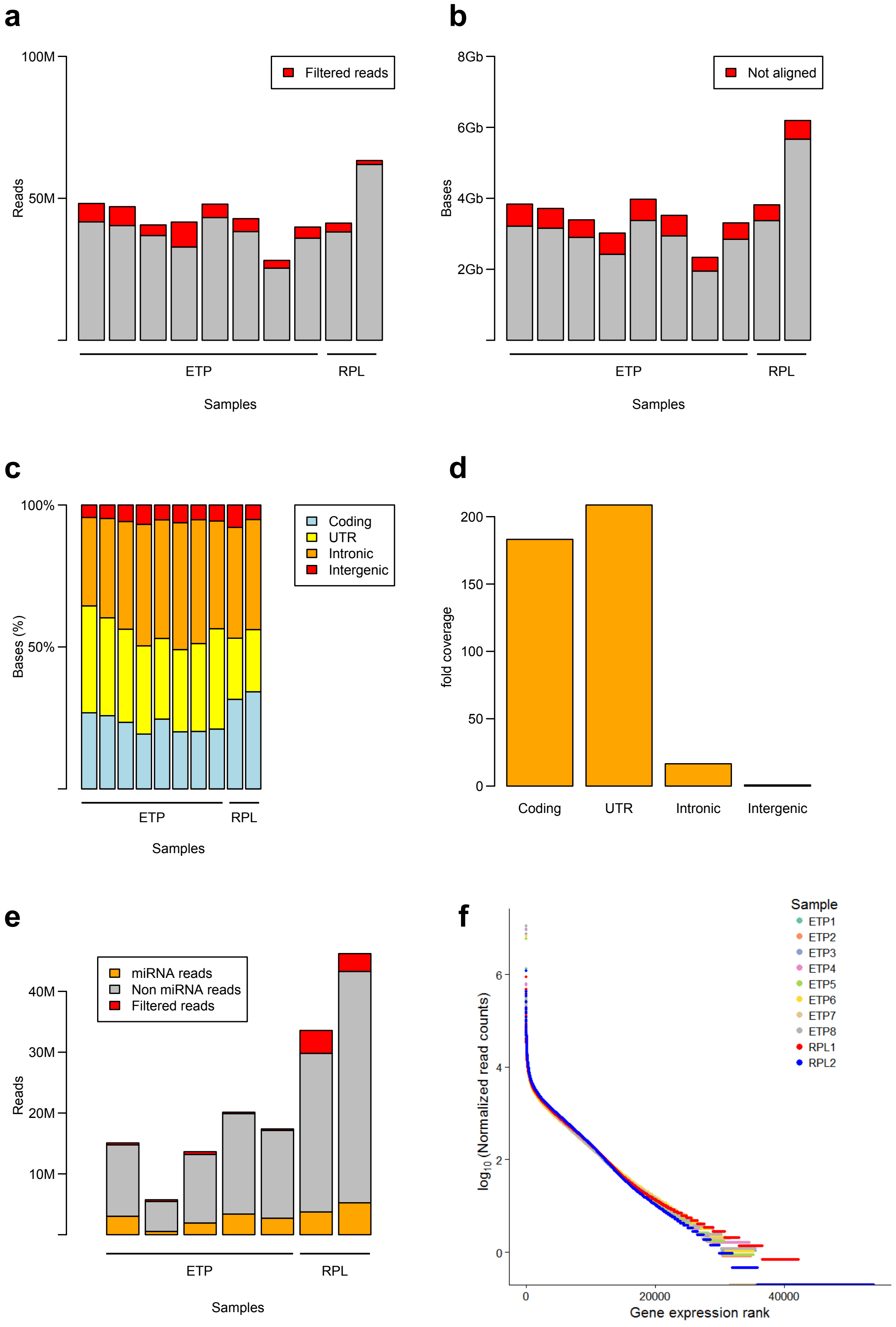

Supplementary Figure S1. RNA-Seq and small RNA-Seq metrics for the analyzed placental tissue samples. (a) Total count of RNA-Seq read pairs for n=8 1st trimester placenta samples from electively terminateted pregnancies (ETP) and n=2 samples from recurrent pregnancy loss (RPL); excluded reads for not matching the filtering criteria (representing rRNA, mtDNA or adaptor sequences and sequences containing homopolymer stretches) are highlighted in red. **(b)** Alignment efficiency; the bars represent the total length of all filtered reads per sample (in Gb) and the sequence fraction unmapped to the human genome reference (Ensembl v67; GRCh37.p7) is shown in red. **(c)** Proportions of aligned bases mapping to the functional genomic domains. (**d**) Sequence coverage of the functional genomic domains estimated for the full dataset across samples; expressed as the mean coverage per bp. *Coding*, protein coding; *UTR*, untranslated regions; *intronic*, introns of multi-exon genes; *intergenic*, regions not annotated as genes. **(e)** Small RNA-Seq read counts for 5 ETP and 2 RPL samples; proportion of reads excluded in quality filtering is shown in red, proportion of non-miRNA reads is shown in gray and miRNA reads in orange. **(f)** Distribution of normalized read counts for genes ordered by expression in each sample.

Supplementary Figure S2.

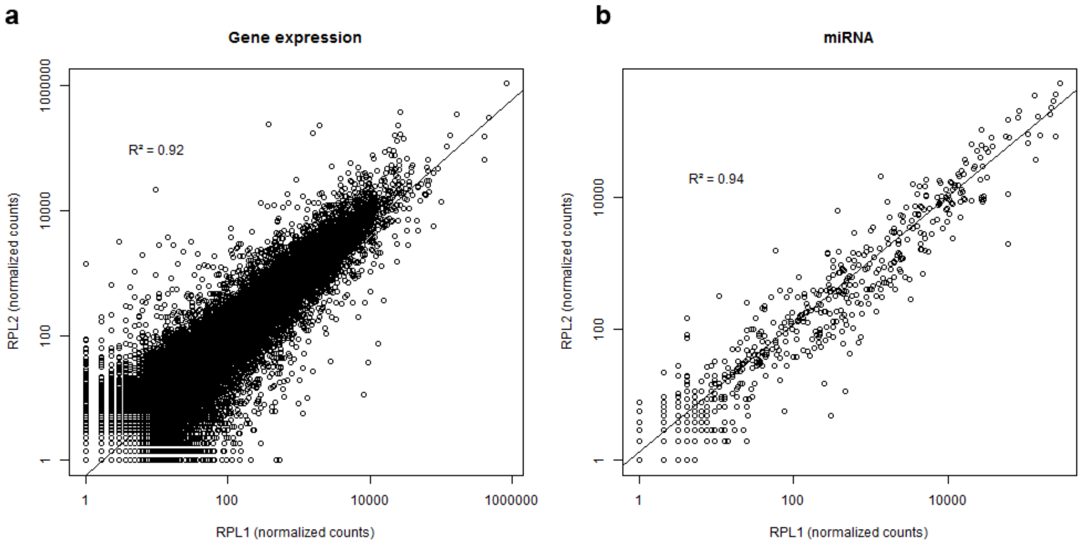

Supplementary Figure S2. High correlation between the transcriptomes of recurrent pregnancy loss placental samples RPL1 and RPL2 in the **(a)** RNA-Seq and **(b)** miR-Seq datasets.

Supplementary Figure S3 (*see legend on the next page*)

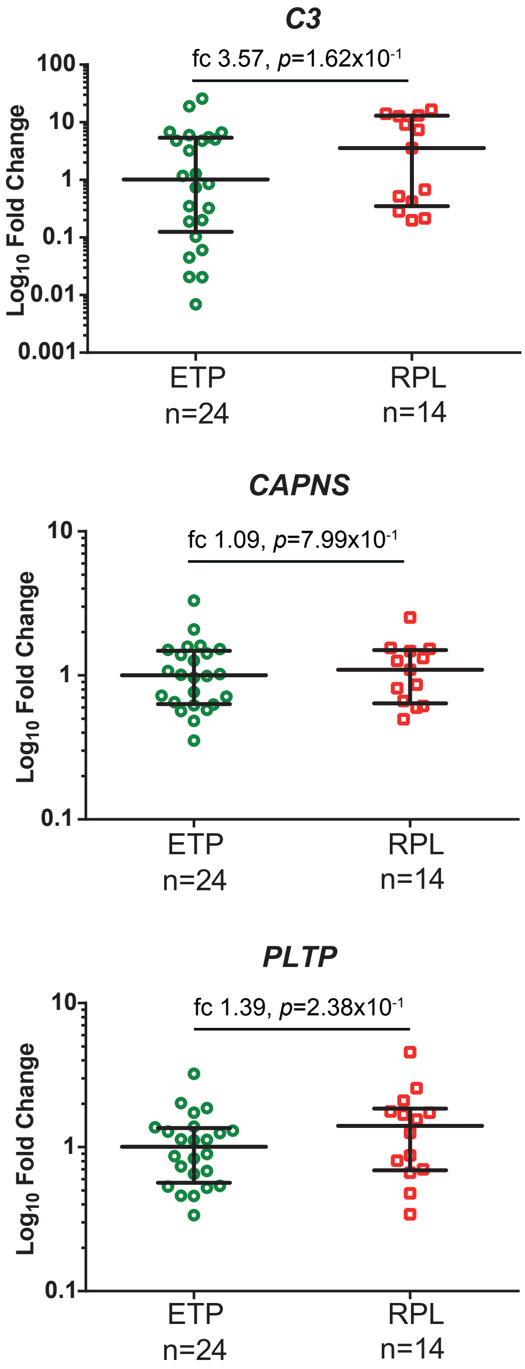

Supplementary Figure S3. Taqman RT-qPCR analysis of differential expression of *C3, PLTP* and *CAPNS1* genes in the placental tissues isolated from the cases of recurrent pregnancy loss (RPL; n=14) compared to electively terminated uncomplicated pregnancies (ETP, n=24). Two RPL samples originally used to generate the discovery RNA-Seq dataset, the rest of the 12 RPL samples and all 24 ETP samples included only for validation experiments. The median expression level of the ETP group was selected as a calibrator and the respective relative mRNA expression levels are shown on logarithmic scale. Dots represent data of each patient; the bars denote median and the 25th and 75th percentiles. Fold change (*fc*) was calculated as the difference of mean relative expression value of the RPL compared to the ETP group; *P*-values were estimated by Wilcoxon test.

C3, complement component 3; *PLTP, phospholipid transfer* protein; *CAPNS1*, *calpain; small subunit 1*

Supplementary Table S1. Samples utilized for Taqman RT-qPCR and clinical characteristics of respective pregnancies

	ETP c(n=24)	RPL cases (n=12)
1st trimester chorionic villi	0	2
1st trimester placental tissue	24	10
Maternal age (years)	28.5 [18 – 37]	32 [27 – 42]
Paternal age (years)	30 [20 – 42]	33.5 [29 – 45]
Maternal pre-pregnancy BMI (kg/m^2)	20.7 [18.0 – 31.3]	22.3 [20.3 – 34.5]
Nulliparity	5 (21%)	6 (50%)
Gravidity	3.5 [1 – 9]	4 [3 – 8]
Previous pregnancy losses	0	2 [2 – 4]
Previous ETP	1 [0 – 4]	0 [0 – 2]
Gestational age at sampling (days)	62 [35 – 91]	53.5 [42 – 103]

Data are given as median and range, except where indicated differently; nulliparity refers to number of previous childbirth and gravidity to the number of experienced pregnancies.
BMI, body mass index; ETP, elective termination of pregnancy; RPL, recurrent pregnancy loss; n, number; N/A, not applicable

Supplementary Table S2. Genes selected for TaqMan RTqPCR and their Assay IDs.

Gene	Taqman Assay ID	Gene Name	Relevant literature [Ref]
C3	Hs00163811_m1	complement component 3	Implantation; pregnancy complications: miscarriage, preeclampsia, etc [1–5]
CAPNS1	Hs00998426_m1	calpain; small subunit 1	DNA damage, acute allograft rejection [6, 7]
HIST1H1B	Hs00271207_s1	histone cluster 1; H1b	benign/malignant tumors, incl. uterine tumors [8]
HIST1H4A	Hs00747492_s1	histone cluster 1; H4a	cell death, tumors [9]
PLTP	Hs00272126_m1	phospholipid transfer protein	Placental expression; pregnancy complications: intrahepatic cholestasis, gestational diabetes [10, 11]
YWHAZ (reference gene)	Hs03044281_g1	tyrosine 3-monooxygenase/tryptophan 5-monooxygenase activation protein, zeta	Stable across gestation and in different pregnancy complications [12–14]

References for Supplementary Table S2

1. Petitbarat, M. *et al*. Critical Role and Therapeutic Control of the Lectin Pathway of Complement Activation in an Abortion-Prone Mouse Mating. *J. Immunol.* **195**, 5602–5607 (2015).

2. Regal, J.F., Gilbert, J.S. & Burwick, R.M. The complement system and adverse pregnancy outcomes. *Molecular Immunology* **67**, 56–70 (2015).

3. Banadakoppa, M., Chauhan, M.S., Havemann, D., Balakrishnan, M., Dominic, J.S. &Yallampalli, C. Spontaneous abortion is associated with elevated systemic C5a and reduced mRNA of complement inhibitory proteins in placenta. *Clin. Exp. Immunol.* **177,** 743–749 (2014).

4. Chow, W.N., Lee, Y.L., Wong, P.C., Chung, M.K., Lee, K.F. & Yeung, W.S.B. Complement 3 deficiency impairs early pregnancy in mice. *Mol. Reprod. Dev.* **76,** 647–655 (2009).

5. Sugiura-Ogasawara, M., Nozawa, K., Nakanishi, T., Hattori, Y. & Ozaki,Y. Complement as a predictor of further miscarriage in couples with recurrent miscarriages. *Hum. Reprod*. **21**, 2711–2714 (2006).

6. Demarchi, F., Cataldo, F., Bertoli, C. & Schneider, C. DNA damage response links calpain to cellular senescence. *Cell Cycle* **9**, 755–760 (2010).

7. Letavernier, E. *et al.* Critical role of the calpain/calpastatin balance in acute allograft rejection. *Eur. J. Immunol.* **41**, 473–484 (2011).

8. Li, H. *et al.* Mutations in linker histone genes HIST1H1 B, C, D, and E; OCT2 (POU2F2); IRF8; and ARID1A underlying the pathogenesis of follicular lymphoma. *Blood* **123**, 1487–1498 (2014).

9. Yan-Fang, T. *et al.* Molecular mechanism of the cell death induced by the histone deacetylase pan inhibitor LBH589 (panobinostat) in wilms tumor cells. *PLoS One* **10** (2015).

10. Floreani, A. *et al.* Intrahepatic cholestasis of pregnancy: new insights into its pathogenesis. *J. Matern.*

Fetal Neonatal Med. **26,** 1410–1415 (2013).

11. Scholler, M. *et al.* Phospholipid transfer protein in the placental endothelium is affected by gestational diabetes mellitus. *J. Clin. Endocrinol. Metab.* **97,** 437–445 (2012).

12. Murthi,P., Fitzpatrick, E., Borg, A.J., Donath, S., Brennecke, S.P. & Kalionis, B. GAPDH, 18S rRNA and YWHAZ are Suitable Endogenous Reference Genes for Relative Gene Expression Studies in Placental Tissues from Human Idiopathic Fetal Growth Restriction. *Placenta* **29**, 798–801 (2008).

13. Meller, M., Vadachkoira, S., Luthy, D.A. & Williams, M.A. Evaluation of housekeeping genes in placental comparative expression studies. *Placenta* **26**, 601–607 (2005).

14. Kaitu'u-Lino, T.J. *et al.* Stability of absolute copy number of housekeeping genes in preeclamptic and normal placentas, as measured by digital PCR. *Placenta* **35**, 1106–1109 (2014).
